# Sequence-based prediction of protein protein interaction using a deep-learning algorithm

**DOI:** 10.1186/s12859-017-1700-2

**Published:** 2017-05-25

**Authors:** Tanlin Sun, Bo Zhou, Luhua Lai, Jianfeng Pei

**Affiliations:** 1 Center for Quantitative Biology, Academy for Advanced Interdisciplinary Studies, Peking University, Beijing, 100871 China; 2 Beijing National Laboratory for Molecular Science, State Key Laboratory for Structural Chemistry of Unstable and Stable Species, College of Chemistry and Molecular Engineering, Peking University, Beijing, 100871 China; 3 Peking-Tsinghua Center for Life Sciences, Peking University, Beijing, 100871 China

**Keywords:** Deep learning, Protein-protein interaction

## Abstract

**Background:**

Protein-protein interactions (PPIs) are critical for many biological processes. It is therefore important to develop accurate high-throughput methods for identifying PPI to better understand protein function, disease occurrence, and therapy design. Though various computational methods for predicting PPI have been developed, their robustness for prediction with external datasets is unknown. Deep-learning algorithms have achieved successful results in diverse areas, but their effectiveness for PPI prediction has not been tested.

**Results:**

We used a stacked autoencoder, a type of deep-learning algorithm, to study the sequence-based PPI prediction. The best model achieved an average accuracy of 97.19% with 10-fold cross-validation. The prediction accuracies for various external datasets ranged from 87.99% to 99.21%, which are superior to those achieved with previous methods.

**Conclusions:**

To our knowledge, this research is the first to apply a deep-learning algorithm to sequence-based PPI prediction, and the results demonstrate its potential in this field.

**Electronic supplementary material:**

The online version of this article (doi:10.1186/s12859-017-1700-2) contains supplementary material, which is available to authorized users.

## Background

Protein-protein interactions (PPI) play critical roles in many cellular biological processes, such as signal transduction, immune response, and cellular organization. Analysis of PPI is therefore of great importance and may shed light on drug target detection and aid in therapy design [1]. Biochemical assays, chromatography, and similar small-scale experimental methods have long been used to identify novel PPIs, but these only contribute to a low coverage of the whole PPI database due to their poor efficacies [2]. High-throughput technologies, such as yeast two-hybrid screens (Y2H) [3] and mass spectrometric protein complex identification (MS-PCI) [4], have generated copious data, however, they are expensive and time consuming. In addition, these methods may not be applicable to proteins from all organisms and often produce false-positive results [5]. Therefore, high-throughput computational methods are needed to identify PPIs with high quality and accuracy.

Recently, many computational methods have been generated to solve this problem. Of these, some have attempted to mine new protein information, whereas others involved the development of new machine-learning algorithms. For protein information mining, Shen et.al regarded any three continuous amino acids as a unit and calculated the frequencies of those conjoint triads in the protein sequences. They demonstrated that PPIs could be predicted by sequences alone [6]. Several other methods, such as autocovariance (AC) [7] and amino acid index distribution [8] were developed to extract features such as physical chemical properties, frequencies, and locations of amino acids to represent a protein sequence. Considering the high dimensions of the features, dimension reduction techniques have been used. For machine-learning algorithms, support vector machine (SVM) and its derivatives [9, 10], random forest [11] and neural networks [12], have been applied. However, most studies provided only the results of cross-validation, and did not test prediction results using external datasets [6, 10, 13, 14].

Deep-learning algorithms, which mimic the deep neural connections and learning processes of the human brain, have received considerable attention due to their successful applications in speech and image recognition [15, 16], natural language understanding [17] and decision making [18]. Compared to traditional machine learning methods, deep-learning algorithms can handle large-scale raw and complex data and automatically learn useful and more abstract features [19]. In recent years, these algorithms have been applied to bioinformatics to manage increasing amounts and dimensions of data generated by high throughput technique [20–24]. For genome regulation function prediction, for example, Xiong et al. applied a deep neural network model to predict DNA variants causing aberrant splicing. Their method was more accurate than traditional models [25]. The DeepBind model constructed by Alipanahi and colleagues using convolutional networks could predict sequence specificities of DNA- and RNA-binding proteins, and identify binding motifs [26]. Identifying functional effects of noncoding variants is a major challenge in human genetics. DeepSEA developed by Zhou et al. could directly learn a regulatory sequence code from large-scale chromatin-profiling data, enabling prediction of chromatin effects of sequence alterations with single-nucleotide sensitivity [27]. After that, the DnaQ model constructed by Quang and coworkers achieved more than a 50% relative improvement compared to other models for predicting the function of non-coding DNA [28]. For protein function prediction, Spencer et al. used a deep belief network (DBN) to predict protein secondary structures and they achieved an accuracy of 80.7% [29]. Sheng and colleagues improved the prediction accuracy to 84% using deep convolutional neural fields [30]. Heffernan et al.’s algorithm can not only predict secondary structures, but also can predict backbone angles and solvent accessible surface areas [31]. A more detailed summary of the application of the deep learning algorithm in computational biology can be found in a recent review [32].

In this study, we applied Stacked autoencoder (SAE) to study sequence-based human PPI predictions. Models based on protein sequence autocovariance coding achieved the best results on 10-fold cross-validation (10-CV) and on predicting hold-out test sets. The best model had an average accuracy of 97.19% for the whole training benchmark dataset. Various external test sets were constructed and predicted using our model and the prediction accuracies for these ranged from 87.99 to 99.21%. In addition, we trained and tested PPI models on other species, and the results were also promising. To our knowledge, our research is the first to use a deep-learning algorithm for sequence-based PPI prediction, and we achieved prediction performance that surpassed previous methods.

## Datasets

### Benchmark dataset

We obtained the Pan’s PPI dataset from http://www.csbio.sjtu.edu.cn/bioinf/LR_PPI/Data.htm [14]. In this dataset, the positive samples (PPIs) are from the human protein references database (HPRD, 2007 version), with removal of duplicated interactions (36,630 pairs remained). Negative samples (non-interaction pairs) were generated by pairing proteins found in different subcellular locations. The protein subcellular location information was from the Swiss-Prot database, version 57.3, according to the following criteria. (1) Only human proteins were collected. (2) Sequences annotated with ambiguous or uncertain subcellular location terms, such as “potential”, “probable”, “probably”, “maybe”, or “by similarity”, were excluded. (3) Sequences annotated with two or more locations were excluded for lack of uniqueness. (4) Sequences annotated with “fragment” were excluded, and sequences with fewer than 50 amino acid residues were removed due to the possibility that they may represent fragments.

In total, 2,184 unique proteins from six subcellular locations (cytoplasm, nucleus, endoplasmic reticulum, Golgi apparatus, lysosome, and mitochondrion) were obtained. By randomly pairing those proteins with others found in different subcellular locations, along with the addition of negative pairs from [33], a total of 36,480 negative pairs were generated. We removed protein pairs with unusual amino acids, such as U and X to yield 36,545 positive samples and 36,323 negative samples to form the benchmark dataset. The interaction networks and the degree distributions of the positive and negative sample sets of the benchmark dataset are shown in Additional file 1 Figure S1 and S2.

We mixed the positive and negative samples in the benchmark dataset and randomly selected 7,000 pairs (3,493 positive samples and 3,507 negative samples) as a hold-out test set for model validation, the remainder of which formed the pre-training set (33,052 positive samples and 32,816 negative samples). The pre-training set was trained and tested using 10-CV, and the best models were selected to predict the hold-out test set. To test the robustness of the model, a non-redundant test set (‘NR-test set’) was formed by removing pairs in the hold-out test set with a pairwise identity ≥25% to those in the pre-training set. After the network architecture and parameters were selected, we trained with the whole benchmark dataset to construct our final PPI prediction model and used it to predict the external test sets.

### External test sets

We used the following datasets as the external test sets.

1. 2010 HPRD dataset: the 2010 version of the HPRD dataset was downloaded and after removal of pairs common to the benchmark dataset, 9,214 pairs were obtained.
2. 2010 HPRD NR dataset: we removed all pairs in the 2010 HPRD dataset with a pairwise identity ≥25% to those in the benchmark dataset, after which, a total of 1,482 pairs remained.
3. DIP dataset: the 20160430 version released Database of Interacting Proteins (DIP, human) was downloaded. After removal of pairs shared with the benchmark dataset, 2,908 pairs were obtained.
4. HIPPIE dataset: The newly released HIPPIE v2.0 was downloaded. It contains the human PPIs from 7 large databases. The scores of PPIs which were equal or larger than 0.73 was regarded as ‘high quality’ (HQ) data by the authors, while the scores of PPIs which were lower than 0.73 was regarded as ‘low quality’ (LQ) data. After removal of pairs shared with the benchmark dataset, 30074 of ‘high quality (HQ)’ PPIs dataset and 220442 of ‘low quality (LQ)’ PPIs dataset were obtained.
5. inWeb_inbiomap: The newly released inWeb_inbiomap was downloaded. It contains the human PPIs from 8 large databases. We screened out the PPIs with ‘confidence score’ equal 1 as the ‘high quality’ (HQ) data and treated the rest as the ‘low quality’(LQ) data. After removal of pairs shared with the benchmark dataset, 155465 of ‘high quality’ PPIs dataset and 459231 of ‘low quality’ PPIs dataset were obtained.
6. 2005 Martin dataset: this dataset was provided by Pan et al.[14].

Note that the samples in datasets 1–5 were all positive and dataset 6 contained both positive and negative samples. Detailed information on the benchmark dataset and the external test sets appear in Additional file 2: Table S1.

### Datasets from other species

We also trained and tested our models using PPI samples from other species, such as *Escherichia coli*, *Drosophila,* and *Caenorhabditis elegans.* The datasets, all obtained from DIP, were provided by Guo et al. (http://cic.scu.edu.cn/bioinformatics/predict_ppi/default.html) and include:

1. *E. coli-*positive dataset containing 6,954 samples.
2. *Drosophila-*positive dataset containing: 22,975 samples.
3. *C. elegans* positive dataset containing 4,030 samples.

The negative samples from each species were also created by pairing proteins from different subcellular locations, and, in all cases, the number of negative samples was equal to the number of positive samples.

## Methods

### Stacked autoencoder

An autoencoder is an artificial neural network that applies an unsupervised learning algorithm which infers a function to construct hidden structures from unlabeled data. Specifically, it attempts to make output $\widehat{x}$ similar to input x, which is an encoding-decoding process. An SAE consists of multiple layers of autoencoders, which are layer-wise trained in turn, and the output of the former layer is wired to inputs of the successive layer.

Consider a stacked autoencoder with n layers; the encoding process of each layer is represented by:

1 $$ {a}^{(1)}= f\left({z}^{(1)}\right) $$

2 $$ {Z}^{\left( l+1\right)}={W}^{\left( l,1\right)}{a}^{(l)}+{b}^{\left( l,1\right)} $$

And the decoding process is its reverse order:

3 $$ {a}^{\left( n+1\right)}= f\left({z}^{\left( n+1\right)}\right) $$

4 $$ {z}^{\left( n+ l+1\right)}={W}^{\left( n- l,2\right)}{a}^{\left( b+ l\right)}+{b}^{\left( n- l,2\right)} $$

**Fig. 1:**
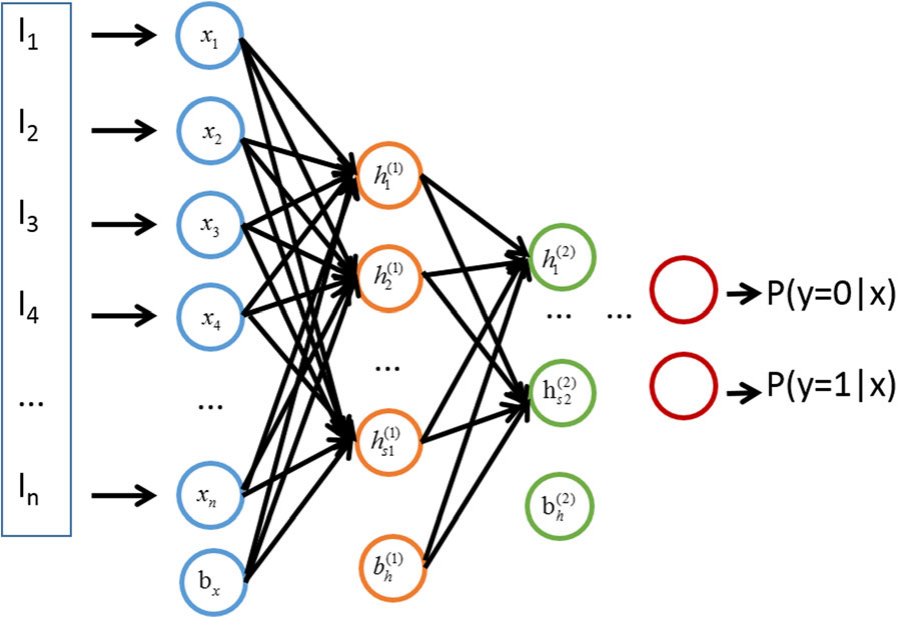
The structure of a stacked autoencoder (SAE)

Where *W* ^(*k*,1)^, *W* ^(*k*,2)^, *b* ^(*k*,2)^, *b* ^(*k*,2)^ represent the weights (*W* ^(1)^, *W* ^(2)^) and Biases (*b* ^(1)^, *b* ^(2)^), respectively, for the kth layer autoencoder, and the useful information is stored in *a* ^(*n*)^. This process may learn a good representation of the raw input after several layers, and we can then link the output to a softmax classifier to fine-tune all the previous parameters using a back-propagation algorithm with classification errors. The structure of a stacked autoencoder is shown in Fig. 1.

Here, we used the SAE DeepLearning Toolbox downloaded from (https://github.com/rasmusbergpalm/DeepLearnToolbox, 20111023). The learning rate and the momentum of the model were the same for both human and other species, while the neurons and layers were tuned and adjusted according to the training set of different species. The detailed information of the training model can be found in Additional file 3.

### Protein sequence coding

We used two methods to code the protein sequences, one is called the autocovariance method (AC) and the other is called the conjoint triad method (CT).

### Autocovariance method

The AC method, which describes how variables at different positions are correlated and interact, has been widely used for coding proteins [12, 34]. The protein sequence is transformed by the following equation:

5 $$ A{C}_{lag, j}=\frac{1}{n- lag}{\displaystyle {\sum}_{i=1}^{n- lag}\left({X}_{i, j}-{\displaystyle {\sum}_{i=1}^n{X}_{i, j}}\right)\times \left({X}_{\left( i+ lag\right), j}-{\displaystyle {\sum}_{i=1}^n{X}_{i, j}}\right)} $$

Where j refers to the j-th descriptor, i is the position of the protein sequence X, ⋅ X_*i*,*j*_ is the normalized j-th descriptor value for i-th amino acid, n is the length of the protein sequence X, and lag is the value of the lag. In this way, proteins with variable lengths can be coded into vectors of equal length (j × lag).

In this study, j is seven (seven physicochemical properties); the names and exact values of these properties are shown in Additional file 4: Table S3. Guo and colleagues [7] selected a value of 30 for the lag and we also used this value. Consequently, the vector contains 210 numbers (7 × 30). The codes of two proteins in a pair were normalized and concatenated as the input to the models.

### Conjoint triad method

The CT method was first proposed by Shen et al. to represent a protein using only its sequence information [6]. First, all 20 amino acids are clustered into seven groups according to their dipole and side chain volumes (Additional file 4: Table S2). Next, each amino acid from a protein sequence is replaced by its cluster number. For example, the protein sequence:

$$ \mathrm{P}=\mathrm{MREIVHIQAG} $$

is replaced by:

$$ \mathrm{P}=3562142411 $$

Then, a 3-amino acid window is used to slide across the whole sequence one step at a time from the N-terminus to the C-terminus.

By calculating the frequency of the combination of each three numbers:

6 $$ \left\{\begin{array}{c}\hfill 111= f1\kern0.5em 121= f8\cdots 177= f337\hfill \\ {}\hfill 211= f2\kern0.5em 221= f9\cdots 277= f338\hfill \\ {}\hfill \begin{array}{l}\kern6em \cdots \\ {}711= f7\kern0.5em 721= f14\cdots 777= f343\end{array}\hfill \end{array}\right\} $$

The protein P is represented by a vector of 343 numbers, all of which are zero except for f276 (356), f89 (562), f13 (621), f149 (214), f71 (142), f158 (424), f23 (241), and f4 (411).

### Evaluation criteria

The performance of the models was evaluated by means of the classification accuracy, specificity, sensitivity, and precision, as defined respectively by:

7 $$ \mathrm{Accuracy} = \frac{TP+ TN}{TP+ TN+ FP+ FN} $$

8 $$ \mathrm{Specificity}=\frac{TN}{TN+ FP} $$

9 $$ \mathrm{Senisitivity}=\frac{TP}{TP+ FN} $$

10 $$ \mathrm{Precision} = \frac{TP}{TP+ FP} $$

Where TP, TN, FP, and FN represents true positive, true negative, false positive, and false negative, respectively.

## Results

### Training

The pre-training dataset was trained with 10-CV, and models with the best performance were selected to predict the hold-out test set. Because the hidden layer and the neuron numbers for each layer of SAE are both critical parameters, we tried different combinations; Details of these combinations are shown in Additional file 5 Figures S3, S4 and Table S4.

Interestingly, for both the AC and CT models (protein sequences coded by AC or CT), one hidden layer was adequate for this task. More specifically, a one-hidden-layer model with 400 neuron numbers using the AC model achieved the best results (average accuracy 96.95%). The overall accuracies of the CT models ranged from 94.2% to 94.5%, with the best average accuracy, 94.52%, achieved at 700 neuron numbers (Table 1). It is worth noting that a one-hidden-layer with a medium neuron numbers was sufficient to train the dataset with relatively high accuracy; more layers and neuron numbers did not improve the predictive power. This phenomenon was also observed by Zeng et al. on predicting the protein binding motif on DNA using a convolutional neural network (CNN) method [23]. This might be due to the specificity of individual task and the nature of the individual dataset.

**Table 1:** The 10-CV training performance of the pre-training models and their prediction performances on test sets

Code	Sen.	Spe.	Pre.	Acc.	Test set acc.	NR-test set acc.
AC	0.9806	0.9588	0.9581	0.9695	0.9682	0.9591
CT	0.9542	0.9367	0.9357	0.9452	0.9447	0.9312

Column 2–5 represent the results of 10-cv with standard deviations ranged from 0.001 to 0.003

Test set acc.: prediction accuracy for the hold-out test set

NR-test set acc.: prediction accuracy for the NR-test set

Then AC and CT models with the best performance with 10-CV were recruited to predict the hold-out test set. The AC model achieved an accuracy of 96.82%, whereas the CT model 94.47%. We removed all pairs in the hold-out test set with ≥25% pairwise identity with those in training set (NR-test set) and used these to confirm the models. The predictive abilities of both models did not decrease appreciably with the NR-test set (Table 1). So, we obtained robust performance on 10-CV training, and for predicting the hold-out and the NR-test sets. Because the AC coding method was superior to the CT coding method for this task, we used AC in the subsequent model construction.

We built our final model with the architecture and parameters of the best trained AC model trained on pre-training dataset. This time the whole benchmark dataset was used for training with 10-CV. We achieved a 10-CV training accuracy as depicted in Table 2, which is one of the best training results compared to the previous methods using the same dataset (Table 3). The Dirichlet allocation (LDA)-random forest (RF) model from Pan et al. yielded the best training accuracy. Regrettably, however, most previous research did not use external test sets to further confirm predictive abilities of their methods, including Pan’s.

**Table 2:** The 10-CV training performance of the final model

Code	Sen.	Spe.	Pre.	Acc.
AC	0.9806	0.9634	0.9627	0.9719

Column 2–5 represent the training results of 10-cv with standard deviations ranged from 0.001 to 0.003

**Table 3:** Comparison of the 10-CV training accuracy to those of previous methods using the same dataset

References	Algorithm	Training Accuracy
[6]	SVM	0.83
[35]	SVM	0.9037
[14]	LDA-ROF	0.9790
[39]	CS-SVM	0.9400
[13]	ELM	0.8480
[10]	SVM	0.9200–0.9740
Our Model	SAE	0.9719

### Prediction of external test sets

Our final model was used to predict the external test sets. We used the newest version of HPRD dataset (2010 HPRD dataset) as one of the external test sets for our model. After excluding the protein pairs that are same in the benchmark dataset, a total of 9,214 PPI were obtained. Our model yielded a prediction accuracy of 99.21%. After the removal of the protein pairs with a ≥25% pairwise sequence identity to those in the benchmark dataset (the 2010 HPRD NR dataset), the prediction accuracy was still high (97.14%) (See more details about the redundancy removal in Additional File 6). We compared our results with Guo’s work. Using the 2009 version of HPRD to test their model, which was based on AC coding and SVM algorithm, Guo et al. achieved a prediction accuracy of 93.59% [35]. Redundancy removal of their test sets resulted in a prediction accuracy of 93.09%. This demonstrated a better prediction capacity of our model.

The 20160430 version of the DIP human dataset (DIP dataset. All PPI pairs in DIP dataset are listed in Additional File 7) was also tested, and this yielded a prediction accuracy of 93.77% (Table 4) for our model. As the training accuracy of the model of Pan et al. was slightly higher than ours, we compared prediction abilities of the two models on external test sets. We submitted the 2010 HPRD, the 2010 HPRD NR, and the DIP datasets to Pan’s online server (http://www.csbio.sjtu.edu.cn/bioinf/LR_PPI), and the returned prediction accuracies on these datasets were 89.15%, 86.70%, and 90.04%, respectively. These values were lower than those obtained with our model (99.21, 97.14 and 93.77%, respectively).

**Table 4:** Prediction performance of the final model on external datasets

Dataset name	Samples	Acc.	Pan et al.’s acc.
2010 HPRD	9214	0.9921	0.8915
2010 HPRD NR	1482	0.9714	0.8670
DIP	2908	0.9377	0.9004
HIPPIE HQ	30074	0.9224	0.8501
HIPPIE LQ	220442	0.8704	--
inWeb_inbiomap HQ	155465	0.9114	--
inWeb_inbiomap LQ	459231	0.8799	--

*HQ* High quality, *LQ* Low quality

Recently, a large number of human PPIs have been verified due to the continually development of the high-throughput technologies. We selected two comprehensive databases that integrated most of the newly-updated PPIs databases (see the Database section) to test our model. The prediction accuracy of the HIPPIE HQ was 92.24% while the prediction accuracy of the HIPPIE LQ was 89.72%. The prediction accuracy of the inWeb_inbiomap HQ was 91.14% while the prediction accuracy of the inWeb_inbiomap LQ was 87.99%. We noticed that our model had better prediction on the HQ dataset than the LQ dataset. We also submitted the HIPPIE HQ dataset to Pan’s server, and the returned prediction accuracy was 85.01%, which was lower than that of our model (92.24%).

Overall, these data suggest that our model, based on SAE, is a powerful and promising tool for the prediction of PPI, especially for the newly released PPIs from the two comprehensive datasets.

A previously generated dataset with 938 positive and 936 negative samples (2005 Martin dataset) [36] has been utilized in a number of previous studies [14, 37–39]. We noticed, however, that most of the previous models used this dataset for training and did not use it for testing. As this dataset is small and has a low coverage on PPI space, the training performance of the previous research using it seems unsatisfying. Notably, Zhang, et al. only used positive samples of the 2005 Martin dataset to test their model and achieved an accuracy of 87.28% [39]. We also tested the 2005 Martin dataset with our model, and we achieved an accuracy of only about 50%, suggesting that the model nearly lost predictive ability (Additional file 8: Table S5). We then tested the positive and negative samples separately and found the prediction accuracy for the positive samples was as high as 94.34% (higher than that of Zhang et al.), whereas for the negative samples, the prediction accuracy was only 6.7%. We also used Pan’s web server to test positive and negative samples from the 2005 Martin dataset, and found that the prediction accuracies were nearly the same as ours (93.52% for positive samples and 5.32% for negative samples). Thus, the model regarded most of the negative samples as positive. We compared the sequence similarities of the positive and negative samples of the 2005 Martin dataset between the positive and negative samples of the benchmark dataset, respectively, and found the unsatisfied result might be due to that the negative samples of 2005 Martin dataset was much similar to the positive samples of the benchmark dataset rather than similar to the negative samples of benchmark dataset (Additional file 8: Table S7).

### Performance on prediction PPI from other species

We also tested the performance of our algorithm with regard to PPIs from *E. coli*, *Drosophila,* and *C. elegans,* with the same training and test data provided by Guo et al. They built their models using SVM with protein coded by AC [35]. Here, we used 5-CV in training which could directly compare with Guo’s result. For *E. coli*, the model was 3 layers and for each layer 420, 500, and 2 neurons were used ([420,500,2]), and this achieved an average training accuracy of 96.05%. For *Drosophila,* the model structure had three layers [420, 300, and 2], and it achieved an average training accuracy of 97.84%. For *C. elegans,* the model structure [420,500,2] achieved an average training accuracy of 97.23%. The detailed training results of our models with Guo et al. ’s training accuracies as comparison are listed in Table 5. It can be seen that for *C. elegans,* we achieved comparable accuracy to Guo et al.’s model, while for *E. coli* and *Drosophila*, our accuracies were higher. Overall, these results demonstrate the power of our algorithm for different species.

**Table 5:** Training performance on PPIs from other species

Species	Sen.	Spe.	Pre.	Acc.	Guo et al.’s acc.
*E. coli*	0.9689	0.9528	0.9518	0.9605	0.9273
*Drosophila*	0.9951	0.9628	0.9616	0.9784	0.9009
*C. elegans*	0.9935	0.9528	0.9508	0.9723	0.9751

Colum 2–5 are the training results of 5-CV with standard deviations ranged from 0.001 to 0.003

## Discussion

Deep-learning algorithms have been used in many fields and their applications in bioinformatics are increasing. However, these powerful methods have not yet been extended to the study of PPI. Thus, in this study, we used a deep-learning algorithm-SAE, in combination with two widely-used protein sequence coding methods AC and CT, to study human PPIs. The performance of our model suggests that the SAE algorithm is robust, and that the AC coding method is superior to CT coding for this task. The training accuracy of our model on the benchmark dataset was comparable to, or higher than, previous models. Our model also had good predictive ability for other external test sets, which were not tested in most previous studies. It is noteworthy that our model gave a satisfying prediction accuracy for a large number of newly verified PPIs. Although Pan et al.’s model achieved the highest training accuracy (97.9%), our prediction accuracies for the three external test sets were significantly better. In addition, we applied our algorithm to train and test PPIs from other species, and performance was promising. Proteins interact with one another through a group of amino acids or domains, so the success of our SAE algorithm may be due to its powerful generalization capacity on protein sequence input codons to learn hidden interaction features.

Although many previous models performed considerably worse on the 2005 Martin dataset, sufficient evidence was not available to explain why this happened. By testing positive and negative samples separately and analyzing sequence similarities between the test and training sets, we found less sequence similarity between the Martin 2005 negative samples and the training negative samples, and we believed that this contributed to unsatisfying prediction accuracy. Because the data were based on unbalanced positive and negative samples, likely the algorithm did not learn many more features than sequence similarity to discriminate between positive and negative datasets (Additional file 9: Table S6, Figure S5).

Considering that only ~2,000 proteins with verified subcellular location were available to construct the negative samples (there were ~9,000 proteins for positive samples), the combined number of protein pairs was insufficient to cover the negative PPI space, prohibiting construction of a reliable PPI prediction model, something also mentioned in Pan et al.’s paper [14]. Our analysis re-emphasizes the need to construct a solid negative dataset with wide coverage of proteins for PPI prediction, in addition to expanding the absolute number of PPI samples for training. This idea agrees with the concept of big data, which emphasizes data complexity besides of data volume. Some consideration has been made for selection of negative samples. For instance, Yu and co-workers proposed that the negative and positive training sets should be balanced to achieve a high-confidence result [40], but Park et al. disagreed, arguing that Yu et al. confused different purposes for PPI analysis [41]. Other groups have tried different methods to build negative data, but did not achieve promising results [6]. We suggest that future work should focus on the construction of a solid and reasonable negative training set, covering negative PPI space as much as possible, to improve the overall prediction accuracy for external datasets.

For protein sequence coding, we used the pre-defined feature extraction methods of AC and CT and the model performed well for predicting PPIs. Either AC or CT has undergone predefined feature selection. With AC coding, physical chemical properties were selected by human knowledge, whereas with CT coding, amino acid classification was made manually. Using pre-defined features for protein function prediction with deep learning algorithm has been common in previous work [29–31]. This deviated somewhat from the essence of deep learning: automatic feature extraction. Future work may focus on developing novel methods for best representing raw protein sequence information.

## Conclusions

In this study, we applied the deep-learning algorithm, SAE, for sequence-based PPI prediction. The best model achieved an average training accuracy of 97.19% on 10-CV training. Its predictive accuracies for diverse external datasets ranged from 87.99% to 99.21%. Furthermore, we trained the datasets from other species, such as *E. coli*, *Drosophila,* and *C. elegans*, and results were also promising. To our knowledge, this research is the first to apply a deep-learning algorithm to sequence-based PPI prediction, and the results demonstrate its potential in this field.

## Additional files

Additional file 1: Detailed description of the benchmark dataset. **Figure S1.** (a) Protein interaction network of the positive samples from the benchmark dataset and (b) negative pairs’ network from the benchmark dataset. **Figure S2.** Degree distribution of the protein interaction network; (a) positive samples from the benchmark dataset, and (b) negative samples from the benchmark dataset. (DOCX 627 kb)

Additional file 2: Detailed information for the benchmark and the external test sets. **Table S1.** Detailed information for the benchmark and the external test sets. (DOCX 14 kb)

Additional file 3: Detailed information about the training model. (DOCX 15 kb)

Additional file 4: Detailed information about protein coding methods. **Table S2.** Classification of amino acids of CT coding method. **Table S3.** Physicochemical properties of amino acid for calculating AC. (DOCX 16 kb)

Additional file 5: Detailed description of the parameter selection. **Figure S3.** The 10-CV training accuracies of the pre-training model in response to increasing numbers of neurons in the one-layer model: (a) AC coding model (AC model) and (b) CT coding model (CT model). **Figure S4.** The 10-CV training accuracies of the pre-training models in response to increasing numbers of neurons in the two-layer models: (a) AC model and (b) CT model. **Table S4.** The 10-CV training accuracies of the three-layer models. (DOCX 174 kb)

Additional file 6: More details about the redundancy removal of the test set. (DOCX 14 kb)

Additional file 7: The PPI pairs in 20160430 version of DIP dataset. (TXT 149 kb)

Additional file 8: Detailed analysis of 2005 Martin dataset. **Table S5.** The predictive performance on the 2005 Martin dataset. **Table S7.** Sequence similarities between the 2005 Martin dataset and the benchmark dataset. (DOCX 13 kb)

Additional file 9: Detailed analysis of the prediction accuracies of the test sets. **Table S6.** Percent of proteins in the test sets having ≥30% sequence identity to those in pre-training/whole benchmark dataset and the prediction accuracy. **Figure S5.** Relationship between the prediction accuracy and the percent of proteins in a test set with ≥30% sequence identity to those in the training set. (DOCX 105 kb)

## Abbreviations

10-CV: 10-fold cross-validation
AC: Autocovariance
CNN: Convolutional neural network
CT: Conjoint triad method
DBN: Deep belief network
FN: False negative
FP: False positive
LDA: The Dirichlet allocation
PPIs: Protein protein interactions
RF: Random forest
SAE: Stacked autoencoder
SVM: Support vector machine
TN: True negative
TP: True positive
