# Supplementary material for: Sequence-based prediction of protein protein interaction using a deep-learning algorithm
Source: BMC Bioinformatics. 2017 May 25;18:277. doi: 10.1186/s12859-017-1700-2 (PMC5445391; doi:10.1186/s12859-017-1700-2)
Supplement: Supplementary file 1 — Detailed description of the benchmark dataset. Figure S1. (a) Protein interaction network of the positive samples from the benchmark dataset and (b) negative pairs’ network from the benchmark dataset. Figure S2. Degree distribution of the protein interaction network; (a) positive samples from the benchmark dataset, and (b) negative samples from the benchmark dataset. (DOCX 627 kb) [file 12859_2017_1700_MOESM1_ESM.docx]

**Additional File 1-Detailed description of the benchmark dataset**

**
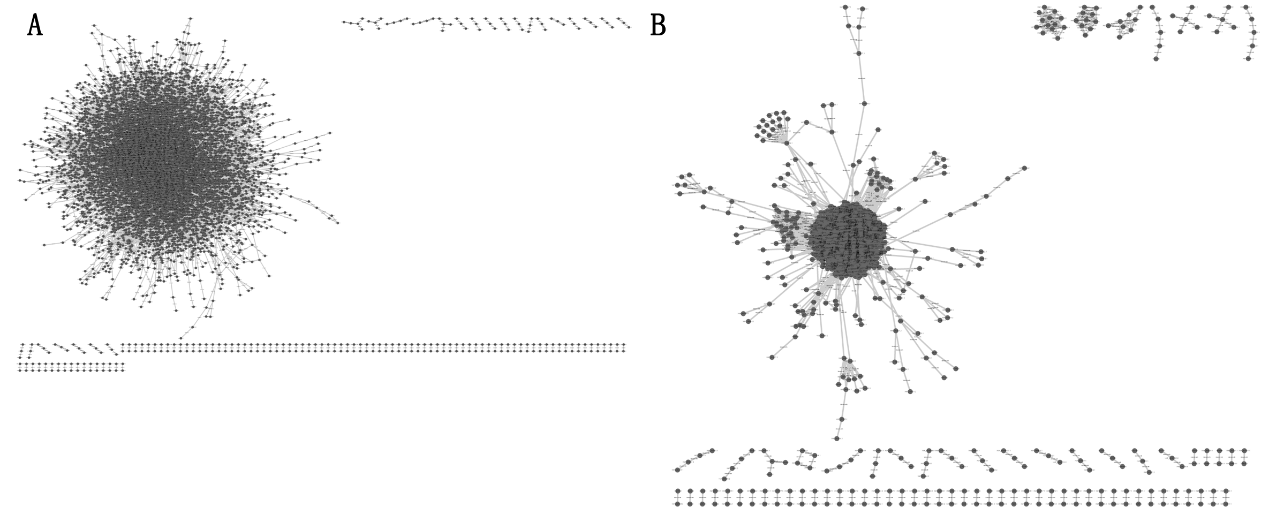
**

**Figure S1.** (a) Protein interaction network of the positive samples from the benchmark dataset and (b) negative pairs’ network from the benchmark dataset.





**Figure S2.** Degree distribution of the protein interaction network; (a) positive samples from the benchmark dataset, and (b) negative samples from the benchmark dataset.
