# Supplementary material for: Sequence-based prediction of protein protein interaction using a deep-learning algorithm
Source: BMC Bioinformatics. 2017 May 25;18:277. doi: 10.1186/s12859-017-1700-2 (PMC5445391; doi:10.1186/s12859-017-1700-2)
Supplement: Supplementary file 2 — Detailed information for the benchmark and the external test sets. Table S1. Detailed information for the benchmark and the external test sets. (DOCX 14 kb) [file 12859_2017_1700_MOESM2_ESM.docx]

**Additional File 2-Detailed information for the benchmark and the external test sets.**

**Table S1.** Detailed information for the benchmark and the external test sets. It is worth noting that the protein pairs from different databases, and even those from different versions of the same database, have different names. We therefore screened them using sequences instead of names, which guaranteed that no duplicated pairs appeared in the training and test sets.

| **Dataset name** | **Train/test** | **Positive/negative** | **Protein** | **Pairs** |
| --- | --- | --- | --- | --- |
| **Benchmark** | train | Positive | 9214 | 33052 |
|  |  | Negative | 2155 | 32816 |
|  | test | Positive | 3510 | 3493 |
|  |  | Negative | 1834 | 3507 |
|  | NR-test | Positive | 1837 | 1366 |
|  |  | Negative | 1530 | 2232 |
| **2010 HPRD** | test | Positive | 5190 | 9214 |
|  | NR-test | Positive | 1542 | 1482 |
| **DIP** | test | Positive | 2369 | 2908 |
| **HIPPIE HQ** | test | Positive | 9420 | 30074 |
| **HIPPIE LQ** | test | Positive | 15720 | 220442 |
| **inWeb_inbiomap HQ** | test | Positive | 9652 | 155465 |
| **inWeb_inbiomap LQ** | test | Positive | 16916 | 459231 |
| **2005 Martin** | test | Positive | 832 | 938 |
|  |  | Negative | 741 | 936 |

HQ: high quality

LQ: low quality
