# Supplementary material for: Sequence-based prediction of protein protein interaction using a deep-learning algorithm
Source: BMC Bioinformatics. 2017 May 25;18:277. doi: 10.1186/s12859-017-1700-2 (PMC5445391; doi:10.1186/s12859-017-1700-2)
Supplement: Supplementary file 3 — Detailed information about the training model. (DOCX 15 kb) [file 12859_2017_1700_MOESM3_ESM.docx]

**Additional file 4-Detailed information about the training model**

Suppose we have a fixed training set $\text{\{（x}^{\text{(1)}},y^{(1)}\text{）},...,(x^{(m)},y^{(m)})\text{\}}$ of m training samples. To train the model, we minimized the objective function, which is defined as below:

$$\begin{matrix} J\text{（}W,b\text{）}=[\frac{1}{m}\sum_{i=1}^{m} J(W,b;x^{(i)},y^{(i)})]+\frac{\lambda}{2}\sum_{l=1}^{n_{i}-1} \sum_{i=1}^{s_{i}} \sum_{j=1}^{s_{i+1}} (W_{ji}^{(l)})^{2} \\ J(W,b;x,y)=\frac{1}{2}\left\|h_{W,b}(x)-y\right\|^{2} \end{matrix}$$

The first term in the definition of J(W,b) is an average sum-of-squares error term. The second term is a regularization term (also called a weight decay term) that tends to decrease the magnitude of the weights, and helps prevent overfitting.

Our goal is to minimize J(W,b) as a function of W and b. We initialized each parameter W^(l)^_ji_ and b^(l)^ _i_ to a small random value and were optimized by stochastic gradient descent with momentum. We did not use regulation strategies since the input number of features were relatively small, the parameter λ was therefore set to 0. For both human and other species, the learning rate was set to 1 and the momentum was set to 0.5. The neurons and layers were tuned and adjusted according to the training set of different species.
