# Supplementary material for: Sequence-based prediction of protein protein interaction using a deep-learning algorithm
Source: BMC Bioinformatics. 2017 May 25;18:277. doi: 10.1186/s12859-017-1700-2 (PMC5445391; doi:10.1186/s12859-017-1700-2)
Supplement: Supplementary file 4 — Detailed information about protein coding methods. Table S2. Classification of amino acids of CT coding method. Table S3. Physicochemical properties of amino acid for calculating AC. (DOCX 16 kb) [file 12859_2017_1700_MOESM4_ESM.docx]

**Additional File 3-Detailed information about protein coding methods**

**Table S2.** Classification of amino acids of CT coding method.

| Number | Amino Acids |
| --- | --- |
| 1 | Ala, Gly, Val |
| 2 | Ile, Leu, Phe, Pro |
| 3 | Tyr, Met, Thr, Ser |
| 4 | His, Asn, Gln, Trp |
| 5 | Arg, Lys |
| 6 | Asp, Glu |
| 7 | Cys |

**Table S3.** Physicochemical properties of amino acid for calculating AC.

| aa. | H_1_ | H_2_ | NCI | P_1_ | P_2_ | SASA | V |
| --- | --- | --- | --- | --- | --- | --- | --- |
| A | 0.62 | -0.5 | 0.007187 | 8.1 | 0.046 | 1.181 | 27.5 |
| C | 0.29 | -1 | -0.03661 | 5.5 | 0.128 | 1.461 | 44.6 |
|  | -0.9 | 3 | -0.02382 | 13 | 0.105 | 1.587 | 40 |
| E | -0.74 | 3 | 0.006802 | 12.3 | 0.151 | 1.862 | 62 |
| F | 1.19 | -2.5 | 0.037552 | 5.2 | 0.29 | 2.228 | 115.5 |
| G | 0.48 | 0 | 0.179052 | 9 | 0 | 0.881 | 0 |
| H | -0.4 | -0.5 | -0.01069 | 10.4 | 0.23 | 2.025 | 79 |
| I | 1.38 | -1.8 | 0.021631 | 5.2 | 0.186 | 1.81 | 93.5 |
| K | -1.5 | 3 | 0.017708 | 11.3 | 0.219 | 2.258 | 100 |
| L | 1.06 | -1.8 | 0.051672 | 4.9 | 0.186 | 1.931 | 93.5 |
| M | 0.64 | -1.3 | 0.002683 | 5.7 | 0.221 | 2.034 | 94.1 |
| N | -0.78 | 2 | 0.005392 | 11.6 | 0.134 | 1.655 | 58.7 |
| P | 0.12 | 0 | 0.239531 | 8 | 0.131 | 1.468 | 41.9 |
| Q | -0.85 | 0.2 | 0.049211 | 10.5 | 0.18 | 1.932 | 80.7 |
| R | -2.53 | 3 | 0.043587 | 10.5 | 0.291 | 2.56 | 105 |
| S | -0.18 | 0.3 | 0.004627 | 9.2 | 0.062 | 1.298 | 29.3 |
| T | -0.05 | -0.4 | 0.003352 | 8.6 | 0.108 | 1.525 | 51.3 |
| V | 1.08 | -1.5 | 0.057004 | 5.9 | 0.14 | 1.645 | 71.5 |
| W | 0.81 | -3.4 | 0.037977 | 5.4 | 0.409 | 2.663 | 145.5 |
| Y | 0.26 | -2.3 | 117.3 | 6.2 | 0.298 | 2.368 | 0.023599 |

H_1_: hydrophobicity; H_2_: hydrophilicity; NCI: net charge index of side chains; P_1:_ polarity; P_2_: polarizability; SASA: solvent accessible surface area; V: volume of side chains;
