# Supplementary material for: Sequence-based prediction of protein protein interaction using a deep-learning algorithm
Source: BMC Bioinformatics. 2017 May 25;18:277. doi: 10.1186/s12859-017-1700-2 (PMC5445391; doi:10.1186/s12859-017-1700-2)
Supplement: Supplementary file 5 — Detailed description of the parameter selection. Figure S3. The 10-CV training accuracies of the pre-training model in response to increasing numbers of neurons in the one-layer model: (a) AC coding model (AC model) and (b) CT coding model (CT model). Figure S4. The 10-CV training accuracies of the pre-training models in response to increasing numbers of neurons in the two-layer models: (a) AC model and (b) CT model. Table S4. The 10-CV training accuracies of the three-layer models. (DOCX 174 kb) [file 12859_2017_1700_MOESM5_ESM.docx]

**Additional File 4-Detailed description of the parameter selection**


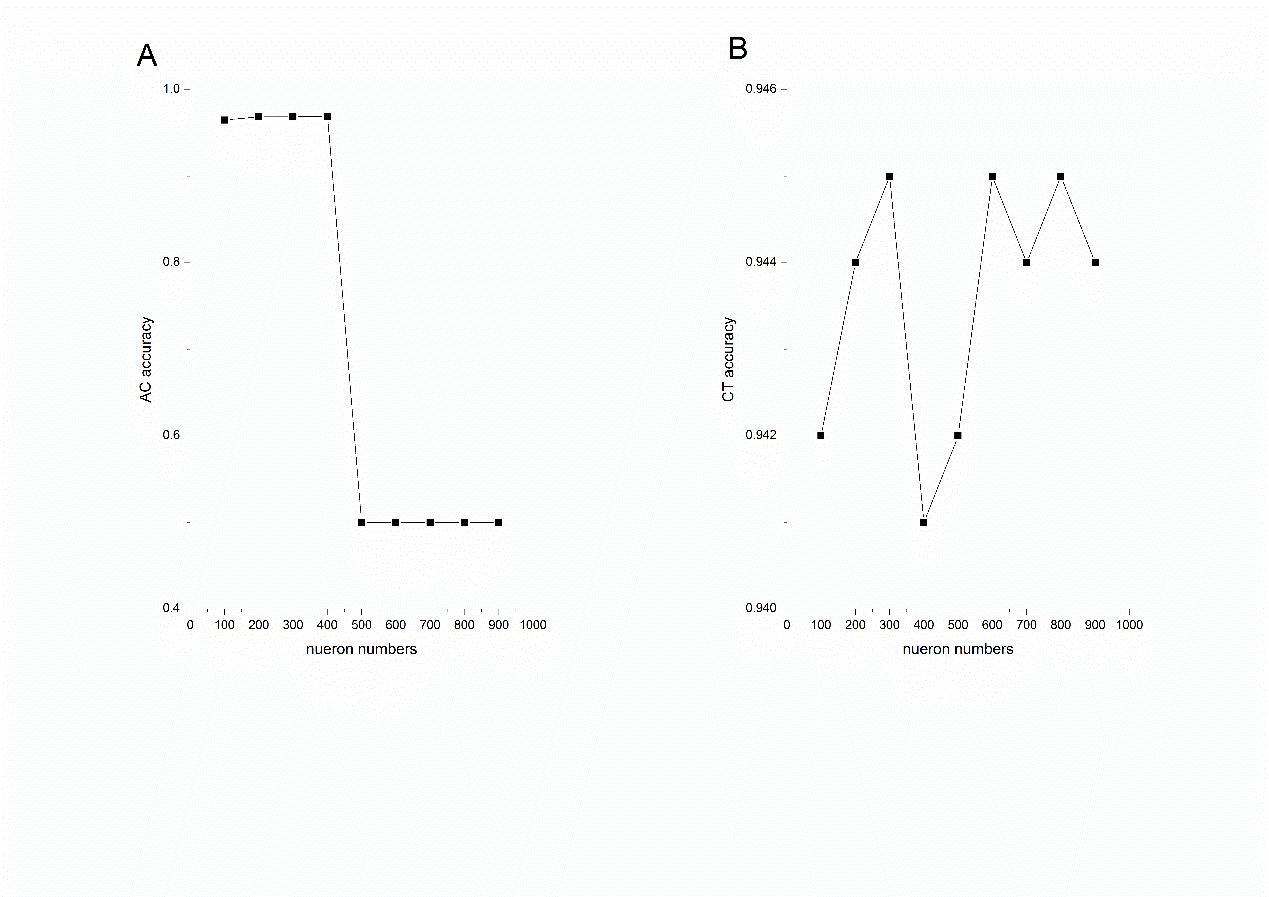
**Figure S3.** The 10-CV training accuracies of the pre-training model in response to increasing numbers of neurons in the one-layer model: (a) AC coding model (AC model) and (b) CT coding model (CT model). The AC model contained 420 input vectors, and the CT model contained 686, with the neuron numbers varying from 100 to 900, and a step length of 100. For the AC model, the highest accuracies were achieved when the neuron numbers ranged from 100 to 400. For the CT model, model accuracies were not very sensitive to the neuron numbers.


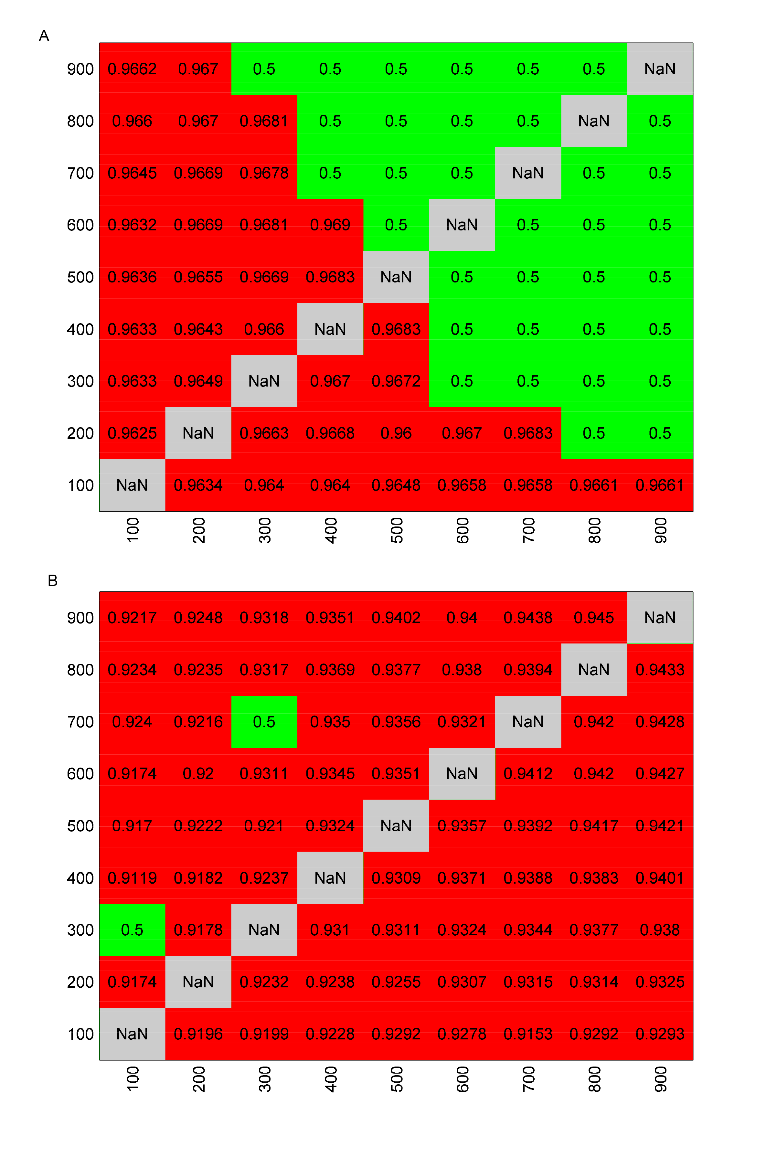
**Figure S4.** The 10-CV training accuracies of the pre-training models in response to increasing numbers of neurons in the two-layer models: (a) AC model and (b) CT model. The AC model contained 420 input vectors, and the CT model contained 686, with the neuron numbers varying from 100 to 900, and a step length of 100. For the AC model, the highest accuracy was achieved when both layers had medium neuron numbers. Notice that 0.5 is not an accurate number, only means that the model loses predictive ability. For the CT model, model accuracies were not very sensitive to the neuron numbers.

**Table S4.** The 10-CV training accuracies of the three-layer models.

| **Layer1** | **Layer2** | **Layer3** | **Accuracy^*^** |
| --- | --- | --- | --- |
| **500** | 400 | 200 | 0.9664 |
| **500** | 400 | 600 | 0.5 |
| **500** | 400 | 800 | 0.4 |
| **600** | 400 | 200 | 0.9622 |
| **600** | 400 | 500 | 0.5 |
| **600** | 400 | 800 | 0.5 |
| **700** | 300 | 200 | 0.5 |
| **700** | 300 | 500 | 0.5 |
| **700** | 300 | 800 | 0.5 |
| **800** | 300 | 200 | 0.5 |
| **800** | 300 | 400 | 0.5 |
| **800** | 300 | 600 | 0.5 |

**^*^**0.5 is not an accurate number, only means that the model loses predictive ability
