# Supplementary material for: Sequence-based prediction of protein protein interaction using a deep-learning algorithm
Source: BMC Bioinformatics. 2017 May 25;18:277. doi: 10.1186/s12859-017-1700-2 (PMC5445391; doi:10.1186/s12859-017-1700-2)
Supplement: Supplementary file 6 — More details about the redundancy removal of the test set. (DOCX 14 kb) [file 12859_2017_1700_MOESM6_ESM.docx]

**Additional File 9- More details about the redundancy removal of the test set**

The test set and training set might share a high number of PPIs that have the same domain, which is a type of redundancy and artificially increase the prediction accuracy. 7 domains listed in Table CL1 widely participate in protein protein interactions. We calculated the number of pairs in the test set that were the ‘domain redundancy’(Suppose pair1 in test set:AB and pair2 in training set:CD; if A and C while B and D or A and D while B and C share the same domain, we consider pair1 as the ‘domain redundancy’ pair). We used the HPRD2010-NR(1482 pairs) dataset as an example and the number of domain redundancy pairs are listed in Table CL1.

Table CL1. Domains and their redundancy pairs

| Domain | Domain redundancy pair |
| --- | --- |
| SH2 | 4 |
| PDZ | 8 |
| SH3 | 7 |
| WW | 5 |
| PTB | 4 |
| FHA | 6 |
| RRM | 6 |

We can see that the number of domain redundancy pairs of all the domains were no more than 10. These results shows that there were not much ‘domain redundancy’ pairs in the test set, and the prediction accuracy will not decrease with the removal of such pairs.
