# Supplementary material for: Sequence-based prediction of protein protein interaction using a deep-learning algorithm
Source: BMC Bioinformatics. 2017 May 25;18:277. doi: 10.1186/s12859-017-1700-2 (PMC5445391; doi:10.1186/s12859-017-1700-2)
Supplement: Supplementary file 8 — Detailed analysis of 2005 Martin dataset. Table S5. The predictive performance on the 2005 Martin dataset. Table S7. Sequence similarities between the 2005 Martin dataset and the benchmark dataset. (DOCX 13 kb) [file 12859_2017_1700_MOESM8_ESM.docx]

**Additional File 5-Detailed analysis of 2005 Martin dataset**

**Table S5.** The predictive performance on the 2005 Martin dataset.

|  | **Sensitivity** | **Specificity** | **Precision** | **Accuracy** |
| --- | --- | --- | --- | --- |
| **AC** | 50.62% | 51.80% | 95.62% | 51.12% |

**Table S7.** Sequence similarities between the 2005 Martin dataset and the benchmark dataset.

| **2005 Martin** | **Benchmark** | **Percent ≥30% sequence identity(%)^*^** |
| --- | --- | --- |
| Positive samples | Positive samples | 96.63 |
| Negative samples | Negative samples | 59.57 |
| Positive samples | Negative samples | 57.37 |
| Negative samples | Positive samples | 96.35 |

*Percent of proteins in the 2005 Martin Positive/Negative dataset that shared ≥30% sequence identity with proteins in the Benchmark Positive/Negative dataset.
