# Supplementary material for: Sequence-based prediction of protein protein interaction using a deep-learning algorithm
Source: BMC Bioinformatics. 2017 May 25;18:277. doi: 10.1186/s12859-017-1700-2 (PMC5445391; doi:10.1186/s12859-017-1700-2)
Supplement: Supplementary file 9 — Detailed analysis of the prediction accuracies of the test sets. Table S6. Percent of proteins in the test sets having ≥30% sequence identity to those in pre-training/whole benchmark dataset and the prediction accuracy. Figure S5. Relationship between the prediction accuracy and the percent of proteins in a test set with ≥30% sequence identity to those in the training set. (DOCX 105 kb) [file 12859_2017_1700_MOESM9_ESM.docx]

**Additional File 6-Detailed analysis of the prediction accuracies of the test sets.**

**Table S6.** Percent of proteins in the test sets having ≥30% sequence identity to those in pre-training/whole benchmark dataset and the prediction accuracy (Figure S5 shows the relationship between them).

| Dataset name |  | Positive/Negative | Protein Percent | | | Accuracy |
| --- | --- | --- | --- | --- | --- | --- |
|  |  |  | Same^1^ | | ≥30% identity^2^ |  |
| Benchmark | Test | Positive | 93.21% | 97.69% | | 96.16% |
|  |  | Negative | 98.58% | 99.23% | | 97.43% |
|  | NR-test | Positive | 91.23% | 95.8% | | 93.41% |
|  |  | Negative | 98.69% | 99.08% | | 97.35% |
|  | S-NR–test^3^ | Positive | 0% | 70.2% | | 85.84% |
|  |  | Negative | 0% | 57.02% | | 25.16% |
| 2010 HPRD | Test | Positive | 77.80% | 98.59% | | 99.21% |
|  | NR-test | Positive | 74.31% | 95.26% | | 97.14% |
| DIP | Test | Positive | 55.29% | 91.00% | | 93.77% |
| 2005 Martin | Test | Positive | 63.29% | 96.63% | | 94.34% |
|  |  | Negative | 14.97% | 59.37% | | 6.7% |
| HIIPIE-HQ | Test | Positive | 63.93% | 90.65% | | 92.24% |
| HIPPIE-LQ | Test | Positive | 45.55% | 80.41% | | 89.72% |
| inWeb_inbiomap-HQ | Test | Positive | 74.87% | 87.98% | | 91.14% |
| inWeb_inbiomap-LQ | Test | Positive | 42.72% | 79.09% | | 87.99% |

^1^‘Same’ means that the proteins are exactly the same

^2^‘For the Test, NR-test, and S-NR–test set, it indicates the percent of proteins in these test sets having ≥30% sequence identity to those in pre-training set, whereas for the 2010HPRD, DIP, 2005 Martin, HIPPIE-HQ, HIPPIE-LQ, inWeb_inbiomap-HQ, inWeb_biomap-LQ test sets, it indicates the percent of proteins in these test sets having ≥30% sequence identity to those in the benchmark dataset.

^3^S-NR-test: the strict non-redundant test set，which was constructed by removing the PPI pair with any protein that has ≥25% sequence identity to any protein in the pre-training set.

**

**

**Figure S5.** Relationship between the prediction accuracy and the percent of proteins in a test set with ≥30% sequence identity to those in the training set.
